# Induced Photonic Response of ZnO Nanorods Grown on Oxygen Plasma-Treated Seed Crystallites

**DOI:** 10.3390/nano8060371

**Published:** 2018-05-26

**Authors:** Waqar Khan, Hafiz Muhammad Salman Ajmal, Fasihullah Khan, Noor Ul Huda, Sam-Dong Kim

**Affiliations:** Division of Electronics and Electrical Engineering, Dongguk University, Seoul 04620, Korea; waqarkyz@gmail.com (W.K.); salman.gikian@gmail.com (H.M.S.A.); fasihullah.khan@dongguk.edu (F.K.); noor.ul.h694@gmail.com (N.U.H.)

**Keywords:** ZnO seed crystals, hydrothermal, oxygen plasma, surface defects, photoluminescence

## Abstract

We examined the influence of O_2_ plasma treatment for the ZnO seed layer (SL) crystallites on the material characteristics of ZnO nanorods (NRs) synthesized by the hydrothermal method. Diode photocurrent and photo-response transient characteristics of the p-Si/n-ZnO-NR heterojunction-based ultraviolet (UV) photodetectors were also examined according to the plasma treatment for the SLs. The superior optical properties of NRs were measured from the photoluminescence by exhibiting 4.6 times greater near-band edge emission when grown on the O_2_-plasma-treated SL. The degree of (002) orientation of the NR crystals was improved from 0.67 to 0.95, as revealed by X-ray diffraction analysis, and a higher NR surface density of ~80 rods/μm^2^ with a smaller mean diameter of 65 nm were also observed by the SL modification using plasma-treatment. It was shown by X-ray photo-electron spectroscopy that this improvement of NR crystalline quality was due to the recovery of stoichiometric oxygen with significant reduction of oxygenated impurities in the SL crystals and the subsequent low-energy growth mode for the NRs. UV PDs fabricated by the proposed SL plasma treatment technique showed significantly enhanced UV-to-dark current ratio from 2.0 to 83.7 at a forward bias of +5 V and faster photo-response characteristics showing the reduction in recovery time from 16 s to 9 s.

## 1. Introduction

Nanostructures using oxide semiconductors such as ZnO, NiOx, SnO_2_, TiO_2_, and CuO have been highlighted recently for various applications such as chemical sensors, photo-electrode materials of dye-sensitized solar cells, non-enzymatic glucose sensors, and ultraviolet (UV) photodetectors (PDs) [1,2,3,4,5]. In particular, high-performance UV PDs have found their various potential applications in monitoring UV radiations in our environments, astronomy, space research, early fire detection, missile technology, and energy harvesting [6,7,8]; therefore, a great amount of research effort has been made on this area in recent decades [5,6,7,8,9,10,11]. Zinc oxide (ZnO), which is one of the most highlighted oxide semiconducting materials, has a direct bandgap of ~3.37 eV and exciton binding energy (BE) of 60 meV at room temperature (RT). These semiconducting oxides are also thermally, mechanically, and chemically stable in harsh environments, exhibiting desirable optical and electrical properties for the active layers in various UV PDs and energy harvesting or optoelectronic devices. Recently, one-dimensional ZnO nanostructures (NSs) such as nanorods (NRs), nanowires, and nanotubes have attracted great attention due to their material strength of large surface-to-volume ratio, which gives rise to huge enhancements in device characteristics in various ways [5,6,7,8,9,10,11,12]. A variety of synthesis methods have been reported to date for the growth of ZnO NRs, which includes vapor-liquid-solid process [5,11], chemical vapor deposition (CVD) [7], pulse laser deposition [13], and hydrothermal method [5,9,12,14]. Among all these methods to grow ZnO one-dimensional NSs, an aqueous solution-based hydrothermal method is known to be attractive due to its low cost, low thermal budget, and simple process architecture for the material synthesis on large-scale substrates [9,12,14].

Following the previous research effort, it could be revealed that different types of defects such as intrinsic defects, surface defects, and other defect states associated with the organic contaminants are very highly populated in the ZnO NRs fabricated by aqueous routes [14,15,16]. These defects in a variety of forms not only degrade the luminescence efficiency but also significantly influence the optoelectronic properties of the NR crystals. To address this issue, many different growth techniques have been examined by modifying the synthesis techniques within the growth process of NRs or optimizing the kinetics of the process. Similarly, various post-growth treatment methods for the NRs were also applied to enhance the structural and optical characteristics [15,16]. From all these attempts, it was understood that the structural quality of seed layer (SL) plays a key role in defect states of the ZnO NSs grown afterwards. Our previous research contribution revealed that even changing the annealing ambient of ZnO seed crystals can greatly affect the morphology, structural, and optical properties of the as-grown NRs [12]. Therefore, the role of SL is obviously essential for the high crystalline quality and vertical growth of ZnO NRs because it provides the following important functions. First, it serves as a nucleation site for initiating the NR growth. It also effectively accommodates the structural mismatch between the substrate and the growing nano-crystallites, and it acts as an electrical channel layer for the charge transfer between the NRs (n-type ZnO) and the substrates during the growth. Therefore, the SL material quality plays a very critical role not only in the crystalline properties of ZnO NR crystals to be grown, but also in the device performance of NR-based PDs, especially the photo-response transient characteristics depending on UV illumination.

In this study, we investigate the evolution of NR crystalline quality pursued by the surface modification of ZnO seed crystallites post-treated by oxygen (O_2_) plasma at different time intervals. For this, we examined the morphological, structural, chemical, and optical characteristics of the seed crystallites and the NRs grown atop the SLs. UV PDs with a p-Si/n-ZnO-NR heterojunction structure were fabricated to verify our analysis on the improvement of NR crystalline quality by the plasma treatment, and important device parameters influenced by the NR material properties were measured and analyzed.

## 2. Experimental Procedure

**Figure 1:**
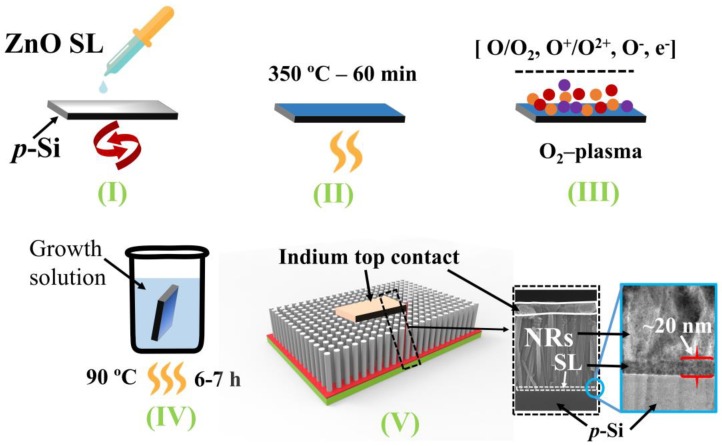
Process flow of the UV photodetector comprising the heterojunction structure of p-Si/n-ZnO NRs grown on SL post-treated by O_2_-plasma.

To fabricate the p-Si/n-ZnO NR-based PDs, we used p^+^-Si (100) substrate (1.5 × 1.5 cm^2^) of a 0.01 Ω-cm resistivity with typical boron doping. The process flow for the fabrication of UV PDs was illustrated in a schematic of Figure 1. All the chemicals used in this work were of a reagent grade purchased from Sigma-Aldrich, Seoul, South Korea and used without any further purification. The NRs were synthesized by the solution-based hydrothermal method as described in the followings. Before starting the device process, all the substrates were first cleaned by acetone, isopropyl alcohol, and de-ionized (DI) water sequentially to remove any dust and organic contaminants. After cleaning the substrates, an aqueous sole-gel solution of zinc acetate dehydrate [Zn(CH_3_COO)_2_·2H_2_O] was prepared with 1-propanol to obtain a final 20 mM concentrate molarity. This SL growth solution was then sonicated for 30 min and spun on the p-Si (100) substrates at 3000 rpm for 30 s in a spin coater. The spin coating process was repeated 10 times to get a nominal thickness of ~20 nm of ZnO SLs as measured by cross-section transmission electron microscopy (bottom-right inset of Figure 1). The seed-grown substrates were post-annealed at 350 °C for 10 min to remove the organic residuals and unwanted residual salts remaining in the SLs. To examine the effect of SL material quality on the material characteristics of NRs to be grown in the next step, we performed O_2_ plasma treatment at RT for the ZnO seed crystallites at a O_2_ flow rate of 100 sccm, a rf power of 100 W, and a process pressure of 50 mTorr for different time intervals of 3, 6, 9, 12, and 15 min. The next hydrothermal growth for the NRs was carried out by immersing the substrates in a growth solution comprising 25 mM equimolar zinc nitrate hexahydrate (Zn (NO_3_)_2_·6H_2_O) and hexamethylenetetramine (HMT, C_6_H_12_N_4_) mixture in 200 mL DI water. After stirring the solution for 25 min to ensure complete mixing, the substrates were placed upside down in a sealable container containing the growth solution and maintained at 90 °C for 6–7 h. Finally, the grown ZnO NRs were washed with DI water and purged with N_2_ gas followed by the moisture removal post-bake at 120 °C for 2 min.

Morphologies of the as-grown NRs on p-Si (100) substrates were analyzed by field emission scanning electron microscopy (FE-SEM) (Hitachi S-4800S, 15 kV, Suwon, Korea). The crystalline quality and preferred orientation of the NRs were investigated by X-ray diffraction (XRD) (D8 Advance spectrometer of Bruker AXS with Cu Kα 0.1540 nm radiation, Suwon, Korea). Atomic force microscopy (AFM, N8-NEOS, Bruker, Suwon, Korea) was carried out in non-contact mode to examine the surface morphology of the as-coated and O_2_-plasma-treated SLs. X-ray photo-electron spectroscopy (XPS) was performed for the analysis of chemical bonding and stoichiometry of the material samples using a PHI 5000 Versa Probe (Ulvac-PHI, Suwon, Korea) spectrometer and a monochromator Al Kα (1486.6 eV) anode (25.0 W, 15 kV, Suwon, Korea). Optical properties of the SLs and NRs were examined by photoluminescence (PL) spectroscopy (MFP-3D Bio, Asylum Research, Suwon, Korea) excited at 266 nm and RT. To observe the influence of O_2_ plasma treatment on the charge transport characteristics of NR crystals, I–V and real-time transient (response and recovery) characteristics of p-Si/n-ZnO NR-based PDs according to UV illumination were measured using a Keithley 2400 source meter (Beaverton, OR, USA, Seoul, Korea) and a light source (254 nm, average intensity of 7 mW/cm^2^) used for deep UV illumination on the heterojunctions. For this measurement, the top electrode was formed by pasting the thin indium contact (~1 mm^2^) on the top of ZnO NRs to provide a good ohmic contact with p-Si substrates, as shown in Figure 1.

## 3. Results and Discussions

**Figure 2:**
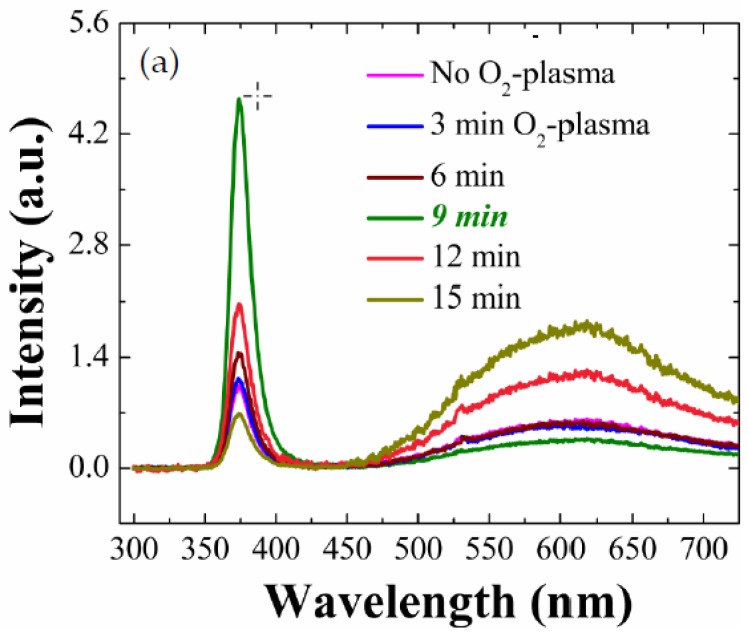

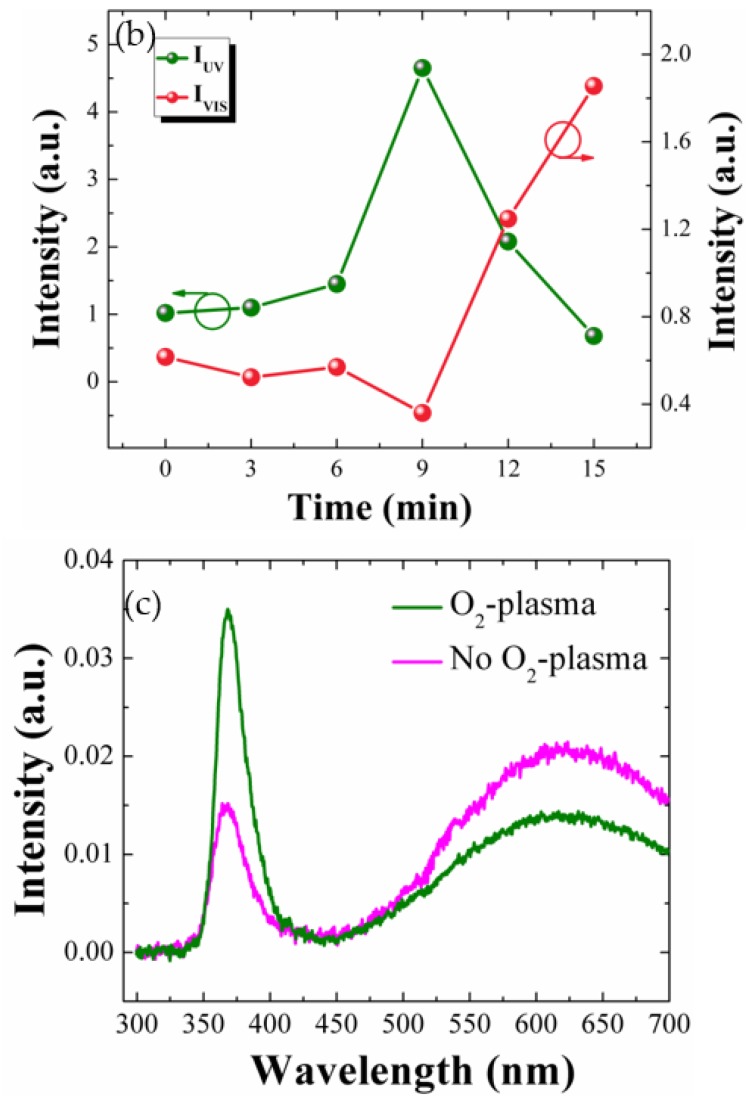
(**a**) RT PL spectra of ZnO NRs grown on SLs plasma-treated for different time intervals; (**b**) I_UV_ and I_VIS_ as a function of time measured from NRs grown on (0, 3, 6, 9, 12, 15 min) O_2_-plasma-treated SLs; (**c**) RT PL spectra of SLs (as-coated and O_2_-plasma-treated for 9 min).

We carried out PL analysis for the ZnO NRs grown on O_2_-plasma-treated SL at various treatment time intervals of 3, 6, 9, 12 and 15 min as depicted in Figure 2a. Luminescence spectra of the as-grown NRs exhibited similar patterns in all cases, where two clear spectral bands of UV emission centered at 3.31 eV (374 nm wavelength) and visible emission centered at 2.03 eV (608 nm) were shown. The UV emission is known to be caused by the band-to-band excitonic recombination which is highly pronounced when the density of defects causing the intra-band transitions is suppressed. The visible emissions, on the other hand, are associated with deep-level emissions (DLEs) and are due to the recombination of photo-generated carriers with various types of intrinsic defects such as oxygen/zinc vacancies, interstitials, and anti-sites [5,14,15,16,17,18]. As shown in the spectra, the UV emissions from the NRs were greatly influenced by the O_2_-plasma treatment for the SL. The comparison of UV emission peaks intensity (I_UV_) and visible emission peaks (I_VIS_) from the NRs as a function of plasma-treatment time is shown in Figure 2b. This result reveals that I_UV_ is increased with the increase of plasma treatment time from 0 min to 9 min; however, I_UV_ starts to fall above a time interval of 9 min. We observed ~4.6 times higher I_UV_ from the NRs grown on the plasma-treated SLs (9 min) than the intensity from the reference sample. This enhancement of I_UV_ is clearly attributed to more improved crystalline quality of the ZnO NRs which is originally due to the improvement of the crystalline properties of the SL materials serving as a nucleation platform for the NRs. The effective volume increase (~30%) of NRs by SL O_2_ plasma treatment can influence the PL emission, but it is thought that the effect is not significant. As shown in Figure 2c, the UV emission from the plasma-treated (9 min) SL crystals is much greater than emission from the as-coated SLs, which can be due to the diffusional flux of atomic oxygens or oxygen radicals from the plasma state into the host material by filling the oxygen vacancies and reduction of organic residuals present on the ZnO SL surface formed during our sol-gel method [19,20].

The origin of each DLE in the visible band is still one of controversial topics because these emissions are due to many complicated intra-band carrier transitions related to various defect states in nanostructure ZnO material. However, in the case of ZnO nanocrystals prepared by solution-based methods such as our hydrothermal growth, hydroxyl groups or oxygenated carbon/hydrogen impurities, which are very difficult to eliminate in general, are known to be the source defects contributing to these DLEs [17,18,19,20]. As we increased the plasma-treatment time up to 9 min, these visible emission peaks from the NR crystals showed reduction in intensity but increased again with the treatment time intervals above 9 min, as depicted in Figure 2b. This degradation of NR optical properties under long-term plasma exposure, as observed in both visible light and UV emissions, can be caused by the over-dose of O_2_-plasma to the SLs where many energetic radicals O* (O^+^, O^−^, O^2+^, and oxygen radical atoms) of high diffusivity deform and/or etch the crystal structure of ZnO SLs [15,19,20]. Figure 2b suggests that, under a plasma power of 100 W used in this study, the optical properties of our NR crystals are improved to the maximum after a treatment time of 9 min and then deteriorated; therefore, the plasma treatment for any further surface analysis and electrical characteristic analysis in this work were carried out for 9 min hereafter.

As shown in Figure 2c, the PL spectra from the SLs showed similar trend with the patterns of the NRs. The UV peak at 3.37 eV (~368 nm) from the O_2_-plasma-treated SLs (9 min) exhibited ~2.4 times higher intensity than that measured from the reference sample. Similarly, the broad-band visible emission from the SLs was also significantly suppressed by the plasma treatment for 9 min.

**Figure 3:**
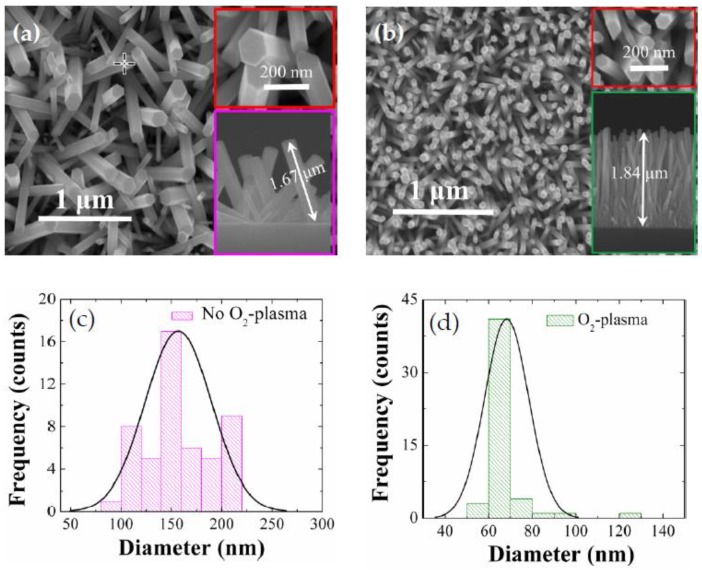
FE-SEM plane views of NRs grown on (**a**) as-coated and (**b**) O_2_-plasma-treated (for 9 min) SLs. Each inset shows the magnified views of the grown NRs. Histograms of diameter distribution for the NRs grown on as-coated and O_2_-plasma-treated (9 min) SLs are also shown in (**c**) and (**d**), respectively.

As shown in the SEM micrographs of Figure 3, the as-grown NRs revealed almost perfect hexagonal wurtzite crystal faces with a growth direction almost perpendicular to the substrates. Measured diameters of the NRs grown on as-coated SLs were fairly widespread ranging from 80 nm to 220 nm with a mean diameter of ~160 nm as shown in the histogram in Figure 3c and an average length of ~1.67 μm. 50 NRs in total were considered for these histogram distribution data of diameters. Aspect ratio and surface density of the NRs in this case were ~11 and ~25 rods/μm^2^, respectively. From the NRs grown on the SL plasma treated for 9 min, we observed a significant change of the NR morphology in diameter, length, aspect ratio, and density, as shown in Figure 3b and Table 1.

More uniform diameter distribution of 50–80 nm was achieved with a smaller mean diameter of 65 nm with a clear reduction in standard deviation of the NR diameter distribution from 7.2 to 5.2, while much higher aspect ratio of ~28 and surface density of ~80 rods/μm^2^ were also observed. The increase of density and reduction of diameter for the NRs are known to be attributed to the increase of nucleation sites caused by the oxidation of excess Zn interstitial atoms segregated in the grain boundary regions into ZnO during the O_2_-plasma treatment [21].

**Figure 4:**
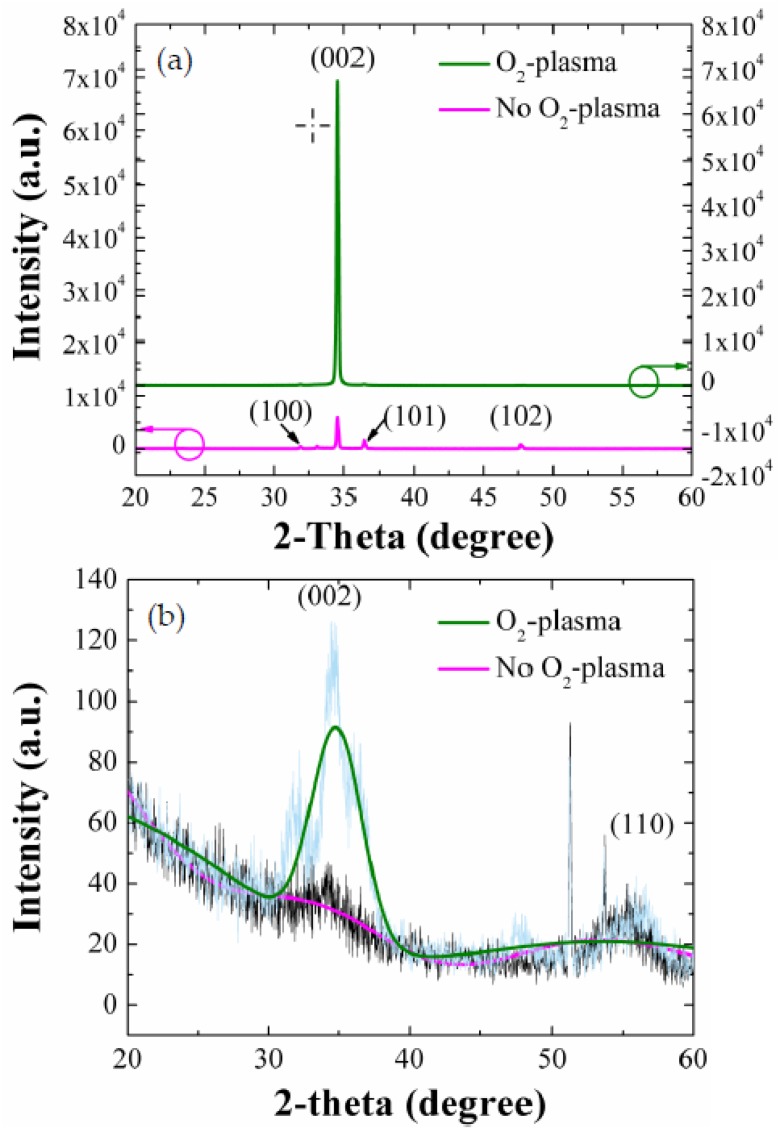
θ–2θ scan XRD patterns for (**a**) ZnO NRs (grown on SLs as-coated and O_2_-plasma-treated for 9 min) and (**b**) SLs (as-coated and O_2_-plasma-treated for 9 min).

To investigate the degree of vertical alignment and crystalline quality more quantitatively, we carried out the XRD measurements for the NRs grown on as-coated and O_2_-plasma-treated (9 min) SLs. As shown in the XRD spectra of Figure 4a, all the principal reflections according to the JCPDS files (card 36-1451) of the wurtzite hexagonal phase of ZnO were recorded from each sample. A very intense peak of (002) at 2θ = 34.5° was observed with no visible reflection from other planes of the NRs grown on the plasma-treated (9 min) SLs. On the other hands, other weak reflections from (100), (101), and (102) planes were also observed at 31.8°, 36.3°, and 47.5°, respectively, with a stronger (002) peak from the NRs grown on as-coated SLs. This reveals that the ZnO NR crystals, when grown on plasma-treated SLs, grow dominantly along c-axis in the vertical direction to the substrate.

As shown from earlier research [22], the ZnO NRs tend to evolve from (002) growth mode of the lowest surface free energy (1.6 J/m^2^) to other crystal plane growth modes such as (100) and (102) (3.4 and 2.0 J/m^2^) when the growth proceeds under a higher energy state, therefore the ZnO film growth can be activated from relative lower surface free energy mode to higher energy mode. As shown in Figure 4b, the plasma-treated SL crystals have a more highly preferred orientation toward (002) than the as-coated SLs. This explains one of the reasons why we have a strong c-axis alignment along the vertical direction to the substrate when the ZnO NRs are grown on the plasma-treated SLs, because each crystalline surface of the SL grain oriented toward a specific direction acts as a nucleus for the growth of crystalline NRs. The degree of (*hkl*) orientation is given by the following relationship [12,23]

F(hkl) = (P(*hkl*) − P_o_(*hkl*))/(1 − P_o_(*hkl*)) (1)

where P(*hkl*) = I(*hkl*)/ΣI(*hkl*), P_o_(*hkl*) = I_o_(*hkl*)/Σ(I_o_(*hkl*), I(*hkl*) is the measured peak intensity from (*hkl*) plane, and I_o_(*hkl*) is the reference peak intensity of (*hkl*) plane given by JCPDS card No. 36-1451. The F(*002*) of the NR crystals grown on O_2_-plasma-treated and as-coated SLs were 0.95 and 0.67, respectively. The enhanced vertical growth of NRs by the SL plasma-treatment can be associated with the suppressed surface contamination and structural defects [15,23] as well as more improved (002) preferred orientation in the SL crystals by O_2_-plasma treatment as discussed earlier.

**Figure 5:**
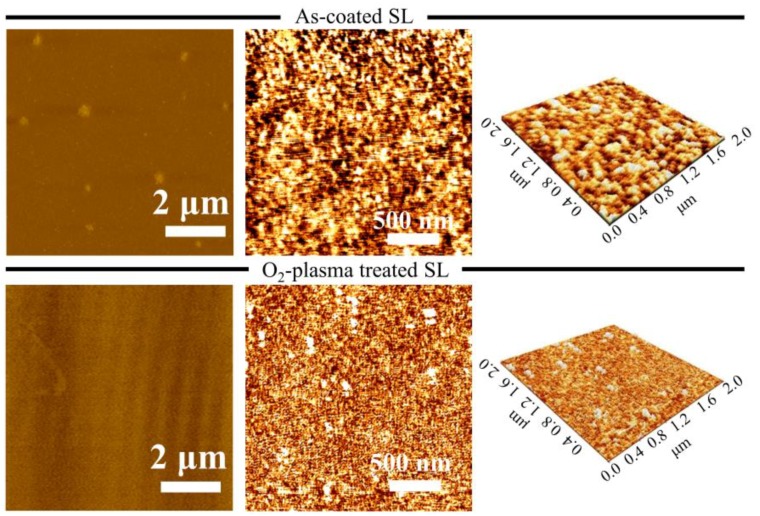
AFM images of as-coated (**top**) row and O_2_-plasma treated SL (**bottom**) row.

AFM was carried out to examine the surface topology of as-coated and O_2_-plasma-treated SLs in an area of 8 × 8 μm^2^ as shown in Figure 5. The surface of O_2_-plasma-treated SL was much smoother and denser with fewer surface voids in contrast with the as-coated SL; the analysis showed a reduction in the root mean square roughness from 0.235 nm (as-coated SL) to 0.151 nm when the SL was plasma-treated. The enhancement in surface smoothness of ZnO thin films by O_2_-plasma treatment is known to be attributed to reduced surface dangling bonds and carbon contaminants, providing oxygen rich seed crystallites for NRs growth [24].

**Figure 6:**
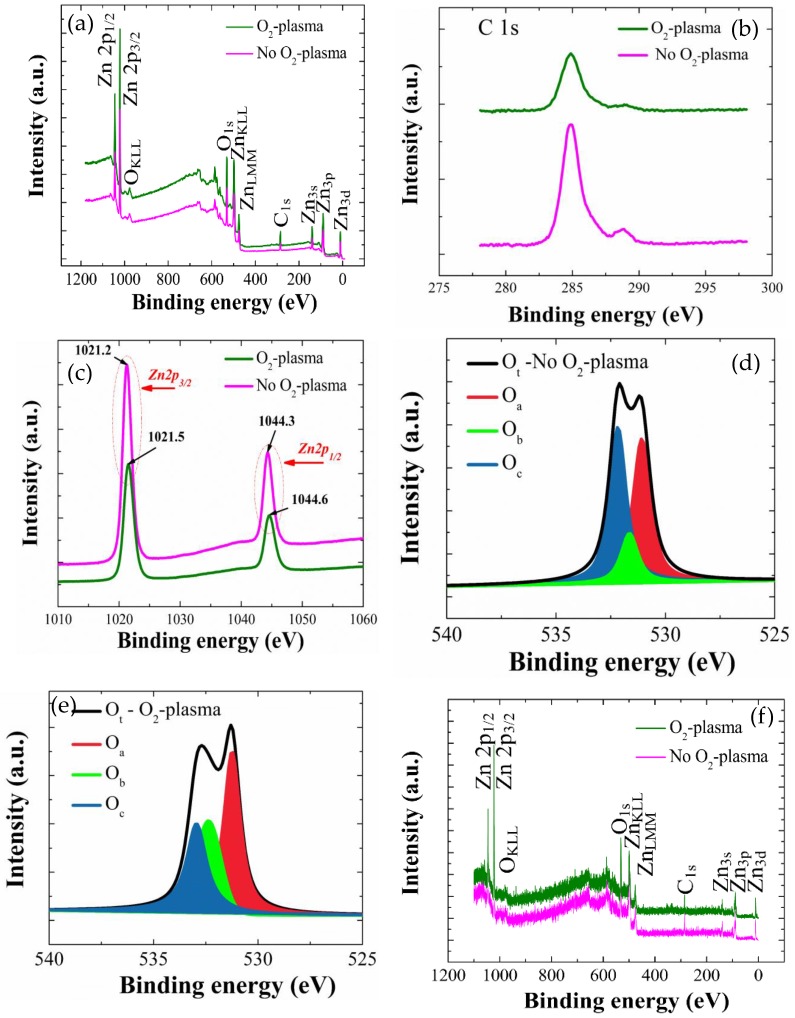

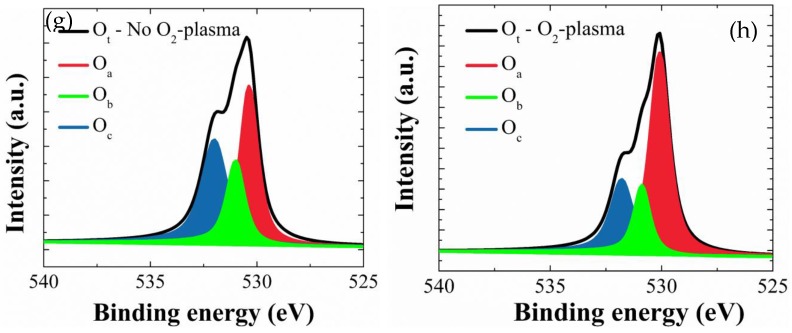
(**a**) XPS wide scan spectra of as-deposited and O_2_-plasma-treated (for 9 min) SLs with high-resolution individual core level spectra of (**b**) C-1s and (**c**) Zn-2p. O-1s spectra obtained from the as-coated and plasma-treated (for 9 min) SLs are also shown in (**d**) and (**e**); (**f**) Wide scan spectra of the ZnO NRs grown on as-deposited and O_2_-plasma-treated (for 9 min) SLs with high-resolution O1-s core level spectra of the NRs grown on (**g**) as-deposited and (**h**) O_2_-plasma-treated (for 9 min) SLs.

We performed XPS analysis in a BE range of 0–1200 eV to investigate the effects of SL plasma treatment (9 min) on the elemental compositions and chemical bindings of each NR and SL crystal. As shown in wide scan spectra of the ZnO SLs of Figure 6a, Zn and O are the main constituents exhibiting various photoelectron peaks of distinct core-levels and spin-orbital splittings as well as Auger peaks, while some traces of carbon were also found in the SL crystals.

First, we analyzed the carbon-containing impurities using the deconvoluted C-1s peaks in a binding energy range of 280–290 eV, as shown in Figure 6b. These C1s peaks are in general attributed to the surface contamination arising from hydrocarbon and carbon oxides during the solution-based synthesis process. More specifically, we could observe clear signal reductions from both major peak from C-C at 284.8 eV and minor peak from COOR or COOH groups centered at 288.8 eV [24] by the SL plasma-treatment. These reductions in carbon impurities are due to the reactions of various oxygen radicals O* in plasma state with the organic residues by forming the byproducts of H_2_O, CO, or CO_2_ which can be evacuated from the chamber during the treatment process [20,24]. The atomic percentages of carbon content were estimated from the SL samples by quantitative analysis, integrating the total area under the C1s curve, and it was shown that the carbon at % was reduced from 21.3% (no plasma treatment) to 16.3% when the SLs were plasma-treated (9 min). The photoelectron peaks from Zn element in the as-coated and O_2_-plasma-treated SLs are shown in Figure 6c with two different core-level Zn peaks of 2p_1/2_ (1044.3 eV) and 2p_3/2_ (1021.2 eV) separated by spin-orbital splitting. From the curve symmetry, peak locations, and spin orbital splitting value of 23.1 eV of the Zn 2p doublet, it can be seen that Zn atoms are present as a Zn^2+^ chemical state in ZnO stoichiometry in all cases [12,25,26]. However, both Zn 2p_3/2_ and Zn 2p_1/2_ peaks from the seed crystals are shifted by 0.3 eV toward higher BE when O_2_-plasma treated. Shifting to higher BE is attributed to the different chemical state of Zn element in the ZnO core matrix. It is thought that the O* radicals diffuse in and take core electrons from the nearest Zn interstitials or stoichiometric Zn present in the ZnO SL matrix, leading to higher Zn atomic charges during the plasma treatment process and ultimately shifting the BE to higher energies [25].

High-resolution O-1s photoelectron peaks from the as-coated and O_2_-plasma-treated SL crystals were depicted in Figure 6d,e, respectively. Gaussian peak deconvolution for the O-1s curve gives three distinct satellite peaks of O_a_, O_b_, and O_c_. O_a_ is a peak at the lowest energy of 530.4 eV which is originated from the O^2−^ ion in the wurtzite structure of hexagonal ZnO [12]. Since O_a_ comes from the stoichiometric oxygen present in the wurtzite structure, we can evaluate the ZnO stoichiometry from ∫O_a_/∫Zn from each SL crystal as summarized in Table 2, where ∫O_a_ and ∫Zn respectively represents the peak curve integration of Oa and Zn in XPS spectra. The percentage contribution of O_a_ in O_t_ (O_t_ = O_a_ + O_b_ + O_c_) can be also obtained by ∫O_a_/∫O_t_, and this contribution of stoichiometric oxygen in total oxygen of ZnO was increased from 37.1% (reference SL) to 61.5% when the SL crystals are plasma treated. This reveals the effectiveness of our O_2_ plasma treatment for the injection of stoichiometric oxygens into ZnO wurtzite structure.

The percentage contribution of each de-convoluted peak was also calculated from the integration of individual curves in O-1s peak and summarized in Table 2. It is common that the ZnO grows in an oxygen-deficient state caused by a high density of oxygen vacancies; however, the O_a_/Zn ratio was increased from 0.61 (reference) to 0.86 when the SLs are plasma treated due to the effectiveness of oxygen injection. The percentage of O_b_, which is the peak at a middle energy of 531.1 eV, was also increased from 11.2% to 21.1%, and this result is thought to be associated with the injected-oxygen participation into O^2−^ ions in the oxygen-deficient region of the ZnO matrix [12,25,26]. Finally, O_c_ is the peak at the highest energy of 532.1 eV, and it has a strong relationship with the chemisorbed oxygen or -OH species on the surface ZnO crystals [12]; therefore, they can evidently cause the surface defects or traps building an increased surface potential barrier or band bending at the ZnO surface. The percentage contribution of O_c_ in O_t_ was significantly reduced from 51.6% (reference) to 17.3% after the treatment, and this result suggests that a huge amount of oxygen-associated surface defects was eliminated by the plasma treatment with the consequent lowering of surface band bending [15,19].

The XPS analysis for the NR crystals grown on SLs was also performed as shown in the spectral responses of Figure 6f. The spectra followed the similar patterns with those from the SL crystals by exhibiting the peaks corresponding to Zn, O, and C. Deconvoluted O-1s peaks from the NRs grown on as-coated and O_2_-plasma-treated SL crystals were shown in Figure 6g,h, respectively. As shown in Table 2, the estimated ∫O_a_/∫Zn ratio representing the O/Zn stoichiometry of the as-grown ZnO NR materials were also significantly increased from 0.70 to 0.91 with a reduction of the O_c_ percentage from 27.8% to 21.2% when the SLs were plasma-treated. Suppose our plasma treatment can effectively eliminate the undesired oxygen-bonded species such as absorbed water, chemisorbed O_2_, and other oxygenated impurities present at the surface or grain boundaries of the SL crystals formed during the growth, we can expect more active intergranular coalescence and grain growth via accelerated diffusion process along grain boundaries. At the same time, a more stable (002) preferential orientation can be achieved from the SL crystals in the post-annealing process. It can be also reasonably assumed that the NR crystals grown on these high crystalline-quality SLs of minimized defects and well-aligned grain orientation tend to preferentially grow perpendicularly to the substrate surface, which is the growth process of the lowest activation energy, thereby producing the high crystalline-quality nanocrystals.

**Figure 7:**
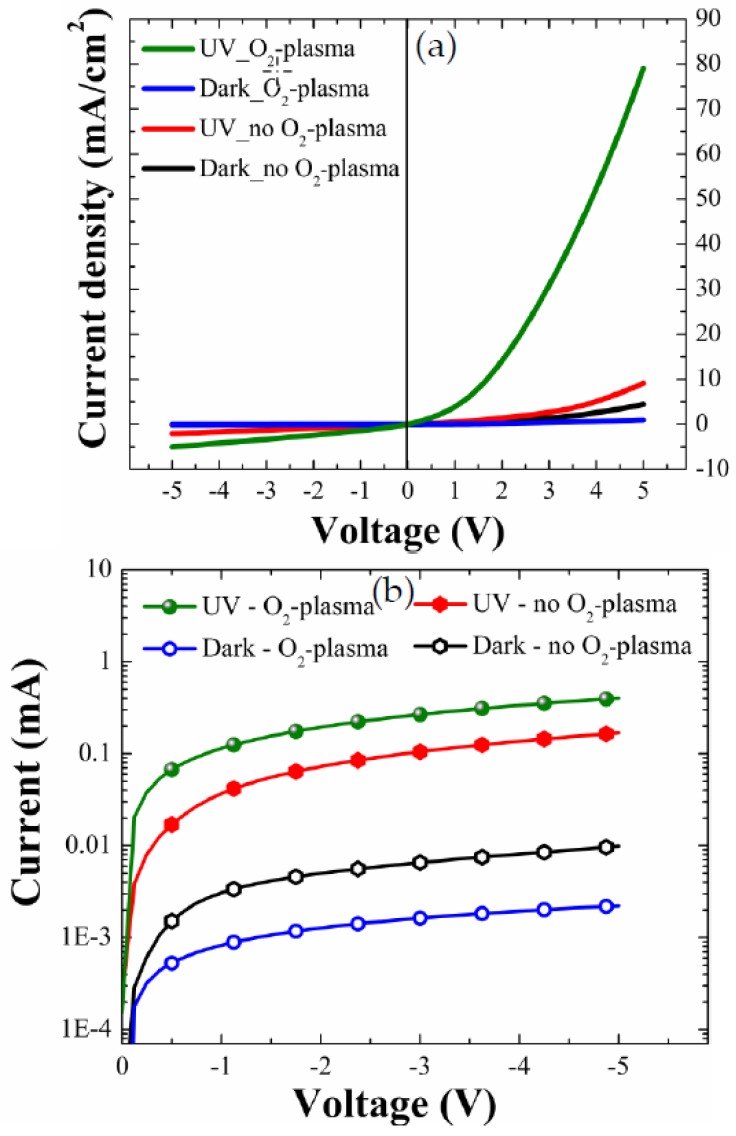
(**a**) Typical diode I–V characteristics in linear scale and (**b**) reverse bias characteristics in log scale, of p-Si/n-ZnO NRs grown on as-coated and O_2_-plasma-treated (for 9 min) SLs under dark and UV illumination conditions.

Based on the results of our material characterizations discussed above, we fabricated the p-Si/n-ZnO-NR heterojunction UV PDs to investigate the effects of improvement in crystalline quality according to the SL plasma treatment on the device characteristics. I–V characteristics of the diodes were first measured across the indium metal contact pasted on the NRs and the other direct probing contact to p-Si. As discussed in our previous report [9], our indium pasting showed a good ohmic characteristic with a specific contact resistance of ~1 × 10^−2^ Ω-cm^2^ (no alloy). Real-time transient responses of the PDs were also measured in a dark chamber of air ambient condition upon the repetitive switching of UV light illumination and turn-off using a light source of 254-nm wavelength and 7-mW/cm^2^ light intensity. Figure 7a shows the typical I–V curves of a well-defined rectifying diode behavior.

The distinctive parameters extracted from I–V characteristics are summarized in Table 3. A great increase of UV-to-dark current ratio (I_UV_/I_Dark_) up to 83.7 was obtained from the PDs with the NRs grown on the plasma-treated (9 min) SL at a forward bias condition of +5 V, while a much lower I_UV_/I_Dark_ of 2.0 was measured from the reference diodes (no plasma treatment). At a reverse bias condition of −5 V, I_UV_/I_Dark_ measured from the diodes with the NRs grown on plasma-treated SL was 182.7, and this ratio was 10.6 times greater than the value measured from the reference diode.

This enhancement of I_UV_/I_Dark_ under forward-bias condition is mainly due to a huge increase of forward-bias photocurrent by the SL plasma treatment. We can find its origin in the enhancement of channel conductivity through the NRs under UV illumination. The chemisorbed oxygen molecule forms a low-conductivity depletion layer near the surface of ZnO NRs which are n-type in nature due to the presence of many oxygen vacancies and Zn interstitials [9,15]. In the dark ambient condition, oxygen molecules are chemisorbed on the surface of ZnO NRs by capturing electrons from the NRs as shown in the process of Equation (2). Under UV illumination, the desorption process (Equation (3)) of chemisorbed O^2−^ ions takes place due to the photogenerated electron-hole pairs from the surface of n-ZnO NRs.

(2) $$O_{2}+e^{-}\to O_{2}^{-}\;(\mathrm{Dark})$$

(3) $$O_{2}^{-}+h^{+}\to O_{2}\;(\mathrm{Under}\;\mathrm{UV})$$

The thickness of the depletion region generated near the NR surface is therefore modulated, so that the NR channel conductivity can be also controlled by the UV irradiation. Therefore, the increase in I_UV_/I_Dark_ by the SL plasma treatment under forward bias condition can be explained by the healing effect of deep-level defects on the NR surface and consequent lowering of surface band bending. In addition to this effect, it is believed that the electric conductivity improvement in the SL region will also contribute to the improvement of the I_UV_/I_Dark_. Effects of morphology (diameter, density, and length) of two different NR structures on the diode I–V characteristics also need to be discussed. Theoretical estimation from two different NRs according to our measurements shown in Table 1 gives that the electrical resistance of NRs grown on O_2_-plasma-treated SL is ~2 times higher than that of the NRs grown on as-coated SL if only geometrical effect is considered. On the contrary, as shown in Figure 7a, the diode in the case of O_2_ plasma-treatment shows a much larger forward-bias current, and thus the effect of NR morphology on the current characteristics of our diodes is thought to be insignificant.

Under reverse-bias condition, the enhancement of I_UV_/I_Dark_ is due to the ~4.5 times lower I_Dark_ of the PD prepared with the SL plasma treatment than that of the reference device, as shown in Figure 7b. This phenomenon was not fully understood, but it can be related to the experimental observation of the huge increase in ZnO sheet resistance after O_2_ plasma treatment [19]. During the plasma treatment process, the oxygen radicals of high diffusivity are expected to fill oxygen vacancies with the diffusion through the ZnO SL reducing the free carrier concentration and the electrical conductivity of the current path between p-Si and n-ZnO NRs. Moreover, the plasma treatment can lead to an enhanced electron depletion effect [20] at the SL-NR interface and the improvement of the electrical connectivity. Before oxygen plasma treatment, a small amount of organic impurities at the SL-NR interface can form the interconnecting bridges across the interface, thereby leading to detrimental charge recombination.

**Figure 8:**
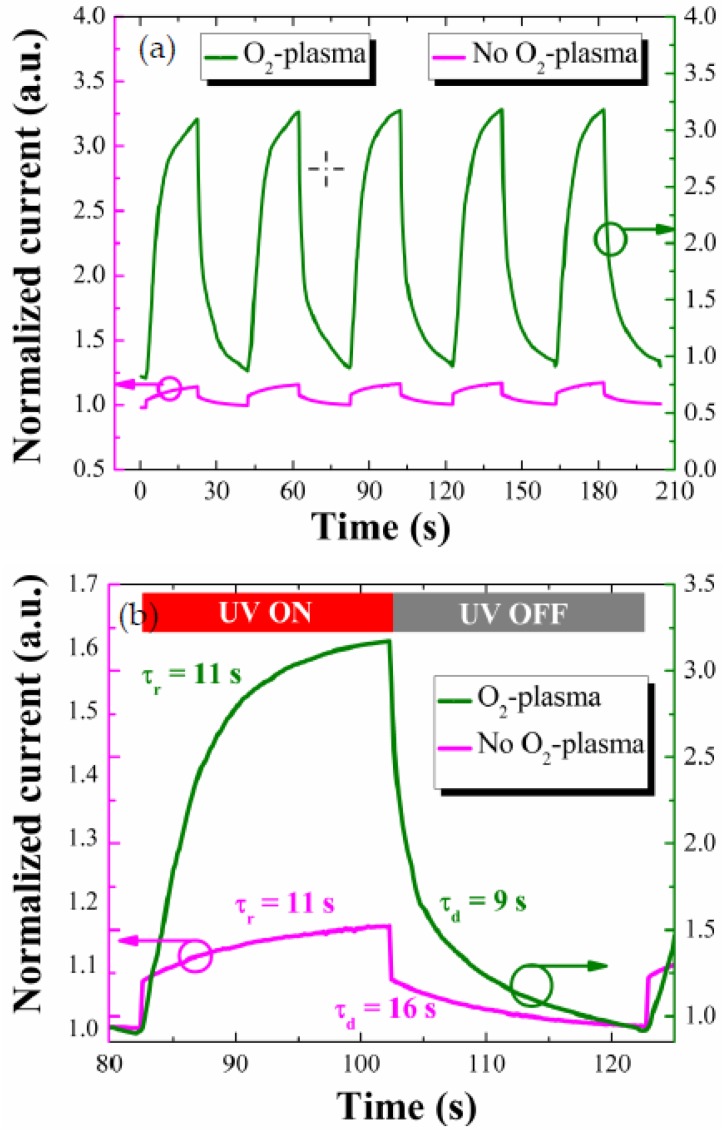
(**a**) Transient characteristics of p-Si/n-ZnO NRs heterojunction PDs grown on as-coated and O_2_-plasma-treated (for 9 min) SLs with (**b**) the expanded views of photoresponse in a single cycle.

One of the important performance parameters for the PDs is the photo-response speed measured from the real-time transients under UV illumination on and off. The photocurrent transients were measured at a forward bias of +5 V with a time lapse of 20 s from the PD devices prepared two different SL conditions, and they all showed a fairly good repeatability as shown in Figure 8a. As shown in the enlarged views of single-cycle transients of Figure 8b, the PDs fabricated with the plasma-treated SL showed faster response speed despite much larger photocurrent upon the UV illumination.

The response time (τ_r_) and the decay (or recovery) time (τ_d_) of this PD were 11 and 9 s, respectively, and the improvement in response speed was significant compared to the reference PD (τ_r_ = 11 s, τ_d_ = 16 s) only in the slower period of recovery. The response and decay time are hereafter defined as time intervals for the photocurrent to rise to 90% of maximum saturation value under UV turn-on and for the photocurrent to fall off by 90% from maximum value under UV turn-off, respectively. Overall transient speed of our PD devices depends on the kinetics of O_2_ adsorption and desorption processes on the ZnO NR surfaces depending UV illumination. However, an improvement in transient speed was obtained by the plasma treatment only from the recovery process which is deeply associated with the O_2_ desorption. From our PL analysis, the UV emission was greatly enhanced with the SL plasma treatment. This suggests that the incorporation of O* into SL crystal does suppress the deep-level defect states on the ZnO NR surface, thereby lowering the surface band bending as discussed in our XPS analysis. When the surface band bending is lowered, the electron diffusional flux toward the NR surface region to produce adsorbed O_2_ ions can be exponentially increased with the reduction of electron energy barrier. If the recovery process is controlled by the kinetics of this electron diffusion and the resulting O_2_ adsorption, we can expect improvement of recovery speed from the proposed SL plasma-treatment technique.

## 4. Conclusions

It was shown that the improvement of ZnO SL material quality was achieved in every aspect of structural, optical, and electrical property examined in this study by the surface post-treatment method utilizing O_2_ plasma. We also found that atomic oxygens or oxygen radicals in the plasma were effectively introduced into the SL surface during the treatment, thereby restoring the stoichiometry of ZnO SL crystals and minimizing the various defects such as oxygen-containing impurities and oxygenated carbons arising from the growth solution. ZnO NR crystals grown on these improved SL crystals showed remarkable improvement in material characteristics in terms of enhanced (002) degree of growth orientation, higher UV emission with the reduced emission from the deep-level states, restoration of O/Zn stoichiometry, and suppression of various intrinsic defects. By growing the n-ZnO NR heterojunction structures on p-Si substrates, we investigated the effects of SL O_2_-plasma treatment on the device characteristics of UV PDs. Great increases in I_UV_/I_Dark_ ratios under both forward and reverse bias modes were obtained due to the enhancement of NR channel conductivity under UV illumination and the suppression of I_Dark_ by the SL surface modification using the plasma treatment. The PDs fabricated with the plasma-treated SL showed much faster response speed especially in the recovery process upon the UV turn-off. This enhancement of response speed is due to the accelerated O_2_ desorption process by suppression of the deep-level defect states on the ZnO NR surface through the SL plasma treatment.

## Figures and Tables

**Table 1:** Summary of morphology statistics measured from the FE-SEM images of ZnO NRs grown on as-coated and O_2_-plasma-treated SLs (for 9 min).

SL	Diameter Range (nm)	Mean Diameter (nm)	Standard Deviation (nm)	NR Density (Rods/μm^2^)	Aspect Ratio
**No O_2_-plasma**	80–20	160	7.2	~25	11
**O_2_-plasma**	50–80	65	5.2	~80	28

**Table 2:** Percentage values of the XPS O-1s satellite peaks from as-coated and O_2_-plasma-treated (for 9 min) SLs and NRs grown on each SL crystal. Each percentage value was calculated by dividing the curve integration of individual satellite peak by the curve integration of total peak.

Condition		O_a_ (%)	O_b_ (%)	O_c_ (%)	O_a_/Zn
SL	No O_2_-plasma	37.1	11.2	51.6	0.61
	O_2_-plasma	61.5	21.1	17.3	0.86
NR	No O_2_-plasma	57.4	14.8	27.8	0.79
	O_2_-plasma	65.9	12.7	21.2	0.91

**Table 3:** Summary of the measured UV-to-dark current ratios at bias conditions of +5 and −5 V from the p-Si/n-ZnO-NR heterojunction PDs.

SL Condition	Parameters	Forward Bias (+5 V)	Reverse Bias (−5 V)
Reference (No O_2_ plasma)	I_dark_ (µA)	353.4	9.8
	I_UV_ (µA)	729.1	168.3
	**I_UV_/I_dark_**	**2.06**	**17.1**
O_2_ plasma	I_dark_ (µA)	75.7	2.2
	I_UV_ (µA)	6337.0	402.1
	**I_UV_/I_dark_**	**83.7**	**182.7**
